# A deep learning model to enhance the classification of primary bone tumors based on incomplete multimodal images in X-ray, CT, and MRI

**DOI:** 10.1186/s40644-024-00784-7

**Published:** 2024-10-10

**Authors:** Liwen Song, Chuanpu Li, Lilian Tan, Menghong Wang, Xiaqing Chen, Qiang Ye, Shisi Li, Rui Zhang, Qinghai Zeng, Zhuoyao Xie, Wei Yang, Yinghua Zhao

**Affiliations:** 1 Department of Radiology, The Third Affiliated Hospital, Southern Medical University (Academy of Orthopedics, Guangdong Province), Guangzhou, Guangdong China; 2 School of Biomedical Engineering, Southern Medical University, Guangzhou, Guangdong China

**Keywords:** Deep learning, Bone neoplasms, Classification, Multimodal imaging, Computer-assisted diagnosis

## Abstract

**Background:**

Accurately classifying primary bone tumors is crucial for guiding therapeutic decisions. The National Comprehensive Cancer Network guidelines recommend multimodal images to provide different perspectives for the comprehensive evaluation of primary bone tumors. However, in clinical practice, most patients’ medical multimodal images are often incomplete. This study aimed to build a deep learning model using patients’ incomplete multimodal images from X-ray, CT, and MRI alongside clinical characteristics to classify primary bone tumors as benign, intermediate, or malignant.

**Methods:**

In this retrospective study, a total of 1305 patients with histopathologically confirmed primary bone tumors (internal dataset, *n* = 1043; external dataset, *n* = 262) were included from two centers between January 2010 and December 2022. We proposed a Primary Bone Tumor Classification Transformer Network (PBTC-TransNet) fusion model to classify primary bone tumors. Areas under the receiver operating characteristic curve (AUC), accuracy, sensitivity, and specificity were calculated to evaluate the model’s classification performance.

**Results:**

The PBTC-TransNet fusion model achieved satisfactory micro-average AUCs of 0.847 (95% CI: 0.832, 0.862) and 0.782 (95% CI: 0.749, 0.817) on the internal and external test sets. For the classification of benign, intermediate, and malignant primary bone tumors, the model respectively achieved AUCs of 0.827/0.727, 0.740/0.662, and 0.815/0.745 on the internal/external test sets. Furthermore, across all patient subgroups stratified by the distribution of imaging modalities, the PBTC-TransNet fusion model gained micro-average AUCs ranging from 0.700 to 0.909 and 0.640 to 0.847 on the internal and external test sets, respectively. The model showed the highest micro-average AUC of 0.909, accuracy of 84.3%, micro-average sensitivity of 84.3%, and micro-average specificity of 92.1% in those with only X-rays on the internal test set. On the external test set, the PBTC-TransNet fusion model gained the highest micro-average AUC of 0.847 for patients with X-ray + CT.

**Conclusions:**

We successfully developed and externally validated the transformer-based PBTC-Transnet fusion model for the effective classification of primary bone tumors. This model, rooted in incomplete multimodal images and clinical characteristics, effectively mirrors real-life clinical scenarios, thus enhancing its strong clinical practicability.

**Supplementary Information:**

The online version contains supplementary material available at 10.1186/s40644-024-00784-7.

## Introduction

Primary bone tumors (PBTs) are a relatively uncommon disease, yet they rank as the third leading cause of cancer-related deaths among individuals under twenty [1, 2]. Depending on their biological behaviors, PBTs are classified as benign, intermediate, or malignant, which require different treatments [3, 4]. For benign PBTs, intralesional tumor excision or curettage is the most suitable treatment. Intermediate PBTs, due to their propensity for recurrence and destructive nature, often demand more aggressive interventions like wide local excision or adjuvant therapy. Malignant PBTs demand a multidisciplinary strategy involving chemotherapy or radiotherapy alongside wide excision or amputation. However, accurately distinguishing benign, intermediate, and malignant PBTs poses challenges due to their overlapping morphologic and radiographical features [5]. For example, while most lesions exhibiting geographic patterns of bone destruction are benign, occasional malignancies demonstrate this radiographic sign [5]. Misclassification of PBTs can lead to overtreatment of patients with benign PBT and delayed or inadequate treatment for those with intermediate or malignant tumors [6]. Therefore, accurate classification of PBTs is essential to guide therapeutic decision-making and ultimately improves patient outcomes.

Medical image modalities such as X-ray, computed tomography (CT), and magnetic resonance imaging (MRI) play indispensable roles in the initial diagnosis of PBTs [3]. X-rays have the advantages of relative cost-effectiveness, rapid imaging, and high spatial resolution, making them the primary imaging modality for evaluating bone destruction, thus serving as the frontline investigation for evaluating PBTs [7]. CT demonstrates remarkable abilities in detecting matrix mineralization (osteoid or chondroid) and thin periosteal bone formations of PBTs, especially beneficial for lesions located in complex anatomical structures [1, 8]. MRI, on the other hand, has superior abilities in detecting early bone changes, soft tissue masses, and the extent of marrow involvement [8]. According to the National Comprehensive Cancer Network (NCCN) practice guidelines, achieving comprehensive radiographic diagnosis of PBTs requires multimodal images combined with clinical characteristics to provide different perspectives [3, 7]. However, due to factors such as equipment unavailability, ionizing radiation exposure, and high cost, most patients’ medical images are often incomplete (i.e., not all patients undergo multiple imaging examinations at the same time) in the clinical setting. This incompleteness poses significant challenges to the accurate classification of PBTs [8–10]. Therefore, there is a pressing need for tools capable of leveraging patients’ incomplete multimodal images to enhance the classification of PBTs.

Recently, several studies have built deep learning (DL) models based on single-modal images, such as radiographs or MRI, achieving good performance in the classification of PBTs [11–13]. However, the clinical utility of these models remains limited due to their incapability to classify PBT patients lacking specific modal imaging data, leading to issues of selection bias and underutilization of overall imaging information [14]. DL models built on incomplete multimodal images have proven feasible and effective in diagnosing neurodegenerative diseases like Alzheimer’s disease [15, 16]. Zhang et al. [17] proposed a Transformer-based method for incomplete multimodal learning of brain tumor segmentation that is robust to any combinatorial subset of available modalities. We recognize the potential of this Transformer-based method in adapting to classification tasks. Hence, it is worth exploring whether the Transformer-based method can be used to classify PBTs using incomplete multimodal images.

This study aimed to develop a Primary Bone Tumor Classification Transformer Network (PBTC-TransNet) fusion model using incomplete multimodal images from X-ray, CT, and MRI, alongside clinical characteristics, to accurately classify PBTs into benign, intermediate, or malignant categories.

## Methods

### Patient cohort and data collection

**Fig. 1:**
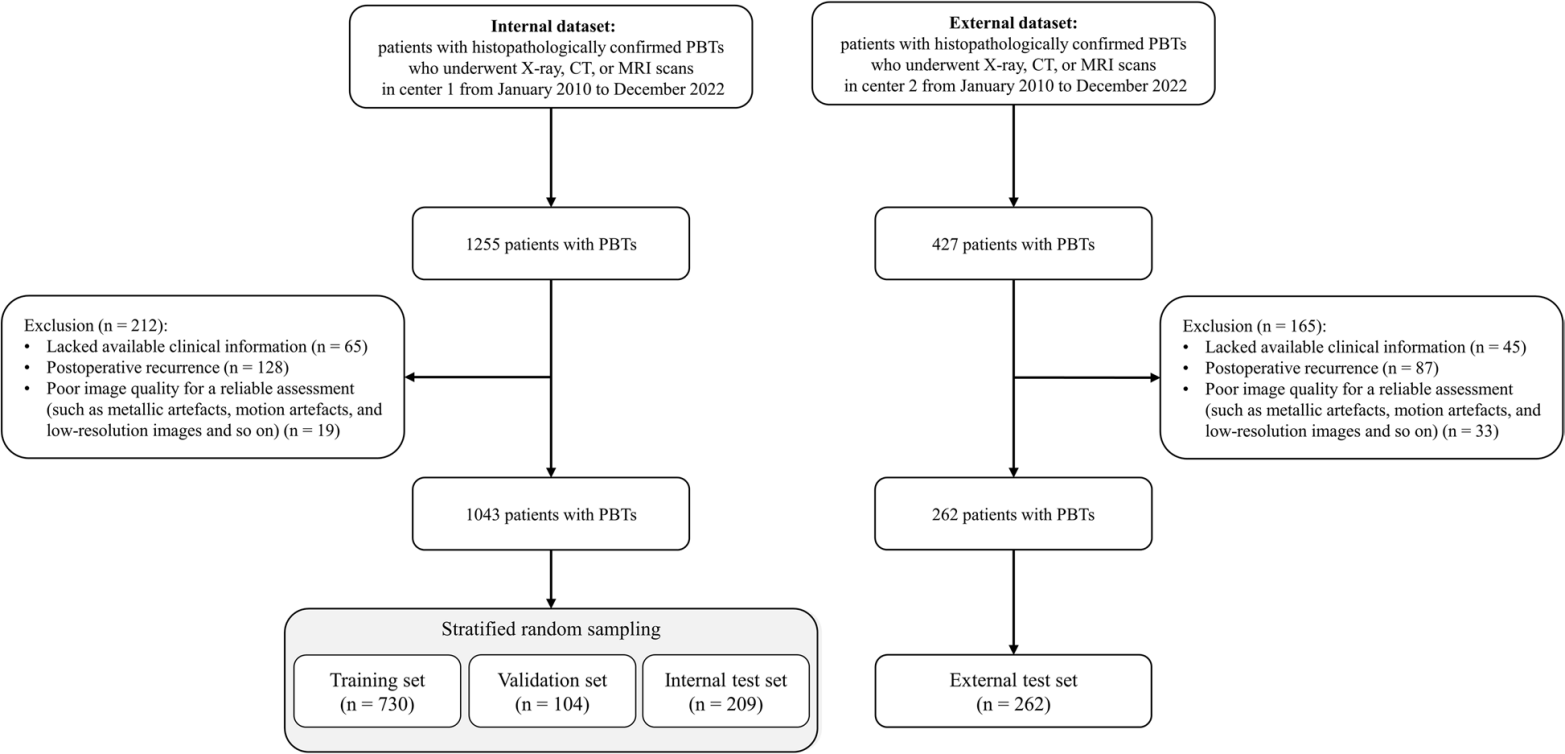
Flow diagram of patient selection. PBTs, primary bone tumors; n, number

This retrospective study enrolled patients diagnosed with PBTs who had undergone X-ray, CT, or MRI scans between January 2010 and December 2022 from two public hospitals (center 1 and center 2) in China. The inclusion criteria encompassed patients (1) who were histopathologically diagnosed with PBTs and (2) who had undergone X-ray, CT, or MRI scans before treatment. Exclusion criteria included patients (1) lacking available clinical characteristics, (2) experiencing postoperative recurrence, and (3) presenting with images of poor quality, as determined by two radiologists (Q.Y. and Y.H.Z.) with 13 and 31 years of experience. Then, these radiologists independently reviewed patients’ histopathological diagnoses via electronic medical records and annotated PBTs as benign, intermediate, or malignant according to the 2020 World Health Organization (WHO) classification [4]. The flowchart of patient selection is summarized in Fig. 1. Finally, a total of 1305 patients from center 1 (the internal dataset, *n* = 1043) and center 2 (the external dataset, *n* = 262) were included. The dataset from center 1 was divided into training, validation, and internal test sets at a ratio of 7:1:2 using the stratified random sampling method with five-fold cross-validation (Appendix S1). The dataset from center 2 was used as an independent external test set. The study protocol was approved by the Institutional Review Boards of center 1 (IRB: 2020-Ethical Review–01) and followed by center 2. On the grounds of retrospective nature, the requirement for written informed consent was waived. This study was conducted according to the Checklist for Artificial Intelligence in Medical Imaging (CLAIM) guidelines (Supplementary Material 2) [18].

Images obtained from various modalities were used as inputs for our models, including X-ray (*n* = 1083); non-enhanced CT (*n* = 821); T1-weighted image (T1WI) (*n* = 622); T2-weighted image (T2WI) (*n* = 577); and T2-weighted image with fat-suppression (T2WI-FS) (*n *= 45). We primarily utilized T1WI and T2WI when available; in cases where T2WI was unavailable, T2WI-FS was employed as an alternative to simulate the real-world medical image distribution. Notably, single view or multiple views (if available) of X-ray images for patients were analyzed (Appendix S2). Additionally, the clinical characteristics of patients, comprising sex, age, location, histopathological fracture, leukocytes, hemoglobin, alkaline phosphatase, as well as symptoms and signs (including redness and hyperemia, swelling, warmth, pain, dyskinesia, and palpable mass), were collected. To gain a deeper understanding of how clinical characteristics influence the model's predictions, we utilized Shapley Additive Explanations (SHAP) values for analysis. SHAP values explain the importance of each characteristic on the model's output. This analysis helps to determine the contribution of each clinical characteristic to the model's predictions [19].

### Data annotation

All images were downloaded and stored as Digital Imaging and Communications in Medicine (DICOM) files at their original size and resolution. Patient-protected health information was deleted from stored files to meet the US (HIPAA), European (GDPR), or Other Relevant Legal Requirements [20]. Two musculoskeletal radiologists (X.Q.C. and Y.Y.S., both with three years of experience) blinded to patients’ histopathologic diagnoses and clinical characteristics, annotated a rectangular region of interest (RROI) covering the whole lesion entity using the ITK-SNAP software (version 3.8.0) [21]. Fifty imaging examinations (X-ray, *n* = 19; CT, *n* = 13; and MRI, *n* = 18) were randomly selected from the internal dataset and re-annotated by the radiologist (X.Q.C.) after a one-month washout period to measure the intra-observer reliability. The inter-observer reliability was calculated by comparing the RROIs of X.Q.C. (first time) and Y.Y.S. The intersection over union (IoU) was calculated to assess inter-observer and intra-observer reliabilities, where IoU greater than 0.5 was considered reliable or reproducible. The results were reviewed by the senior musculoskeletal radiologist (Y.H.Z.) to confirm the final RROIs.

### Development of the deep learning models

**Fig. 2:**
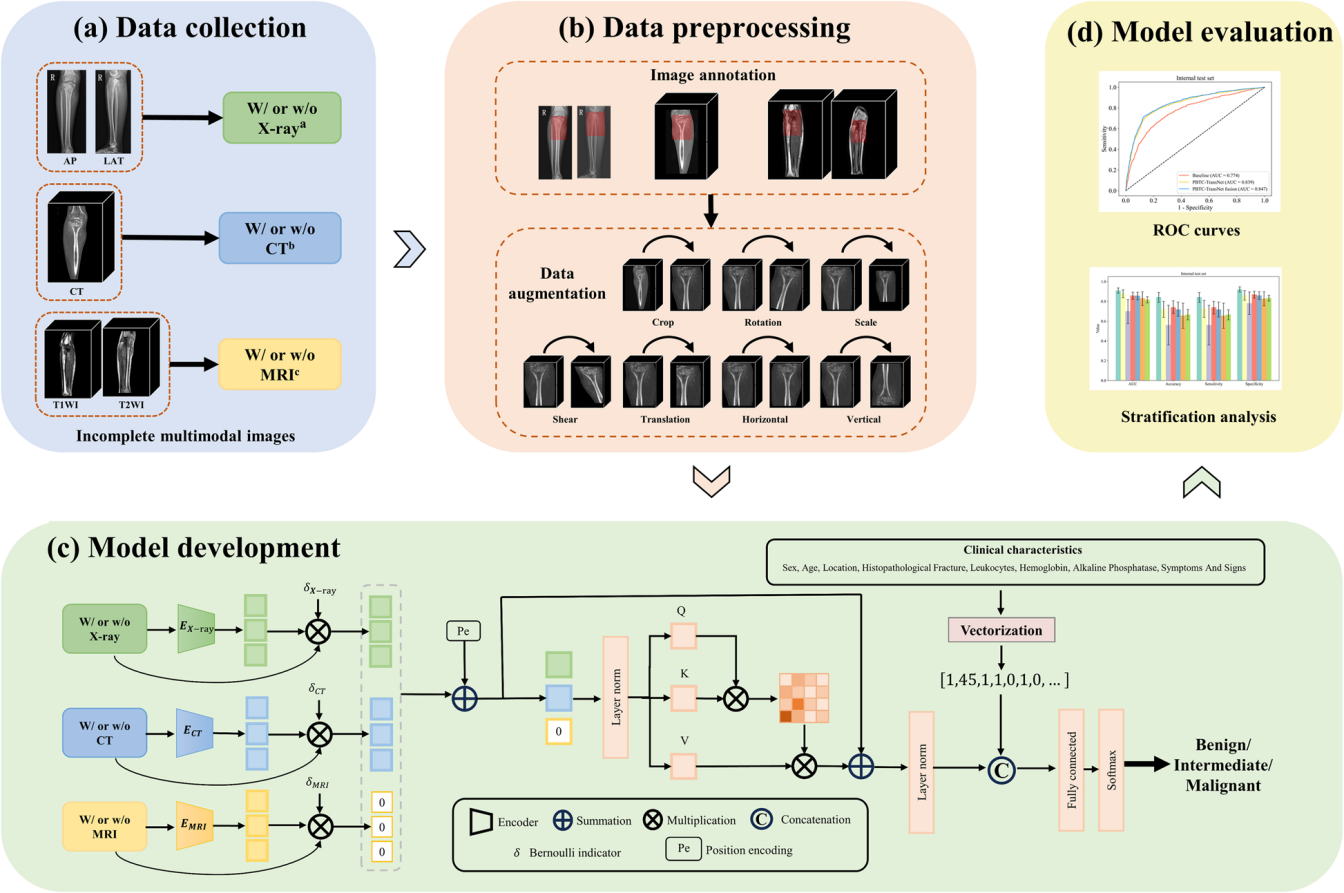
Workflow of this study. **a** Collection of incomplete multimodal images. **b** Preprocessing of the images. **c** The framework of the deep learning models. **d** Assessing the performance of models. ^a^Single view or multiple views of X-ray images are analyzed. ^b^CT includes non-enhanced computed tomography. ^c^MRI includes T1-weighted image, and T2-weighted image or T2-weighted image with fat-suppression. AP, Anteroposterior projection; LAT, lateral projection; ROC, Receiver operating characteristic; PBTC-TransNet, Primary Bone Tumor Classification Transformer Network; W/, with images; w/o, without images. The simultaneous absence of X-ray, CT, and MRI is forbidden

Image preprocessing included intensity normalization and data augmentation. The detailed image preprocessing procedures are described in Appendix S3. We proposed a novel Transformer-based PBTC-TransNet for classifying PBTs as benign, intermediate, or malignant (Fig. 2). The PBTC-TransNet employed three encoders inspired by ResNet [22] to extract modality-specific features. The CT and MRI encoders each consisted of one 3D convolution block and three 3D residual blocks, while the X-ray encoder included one 2D convolution block and three 2D residual blocks (Fig. S1). To simulate real multimodal incomplete scenarios, We employed Bernoulli indicators $\delta \in \left\{0, 1\right\}$ for each modality, where the value of the Bernoulli indicators depends on the presence of the modality. The corresponding Bernoulli indicator was set to zero when the modality was not available. Subsequently, the Transformer was utilized to effectively learn long-range dependencies among different features. Finally, the fully connected layers and Softmax function were used to generate classification results. As a comparison, we developed a Baseline classification model that separately trained models on each available imaging modality, which trained two 3D EfficientNet models for CT and MRI and a 2D EfficientNet for X-ray [23]. The EfficientNet is a powerful CNN architecture, which may provide significant medical image classification potentials while requiring fewer computation resources than other models [24]. Moreover, we used an iterative imputation strategy based on the chained equation forest (Mice-Forest) to impute the missing clinical characteristics (Appendix S4). Then, we integrated the values of clinical characteristics into the final fully connected layer to develop the PBTC-TransNet fusion model. Further details of model development are provided in Appendix S3. All models were implemented using PyTorch (version 1.11.0) in open-source software Python (version 3.8.3) with an NVIDIA RTX 2080Ti GPU of 11 GB memory. The source code is available online (https://github.com/SMU-MedicalVision/PBTC-TransNet).

### Statistical analysis

The Kruskal–Wallis and chi-square tests were used to determine significant differences among clinical characteristics. Classification performance was quantified using the areas under the receiver operating characteristic curve (AUC), accuracy, sensitivity, and specificity, with 95% confidence intervals (CIs) estimated using Bootstrap methods. The micro-average was calculated for AUC, sensitivity, and specificity (Appendix S5). AUC values were compared using the DeLong test, while accuracy was compared using the McNemar test. In addition to evaluating the overall performance, we also stratified the data by age, gender, and time period to assess the model's applicability across different demographic groups. The age stratification was done by dividing the patients into quartiles based on their ages for subgroup analysis. A two-sided *p* value less than 0.05 was considered statistically significant. All analyses were conducted using SPSS (version 23.0, IBM, Armonk, NY) and Python (version 3.8.3).

## Results

### Patient characteristics and datasets

A total of 1305 patients (mean age, 26 years ± 18 [standard deviation]; 512 females) were involved in the study, including 752 benign, 228 intermediate, and 325 malignant cases. There were significant differences among the benign, intermediate, and malignant PBT groups in age, location, leukocytes, hemoglobin, alkaline phosphatase, as well as patient symptoms and signs, including swelling, warmth, pain, dyskinesia, and palpable mass (all* p* < 0.001) (Table 1). No significant difference was observed in the distribution of benign, intermediate, and malignant PBTs between internal and external datasets (*p* = 0.513) (Table S2). The variable distributions of the original and imputed clinical characteristics were similar, indicating that the imputed data can be used for further analysis (Fig. S2). Regarding imaging modalities, on the internal dataset, 850 (81.5%) patients underwent X-rays, 658 (63.1%) underwent CT scans, and 547 (52.4%) underwent MRI examinations. On the external dataset, 233 (88.9%) patients underwent X-rays, 163 (62.2%) underwent CT scans, and 75 (28.6%) underwent MRI examinations (Table 2). Tables S3 and S4 present the distribution of PBT subtypes and imaging modalities among the patients. Excellent inter-observer and intra-observer agreement were achieved for annotating the RROI, with IoUs of 0.839 ± 0.113 and 0.871 ± 0.082, respectively.

**Table 1:** Clinical characteristics of patients in the study

Characteristic	Benign (*n* = 752)	Intermediate (*n *= 228)	Malignant (*n* = 325)	All (*n* = 1305)	*p* value
Age (years)	21 ± 16	31 ± 20	31 ± 19	26 ± 18	< 0.001*
Sex: female	284 (37.8)	100 (43.9)	128 (39.4)	512 (39.2)	0.255
Locations overall					< 0.001*
Torso or head	72 (9.6)	47 (20.6)	77 (23.7)	196 (15.0)	
Extremities	680 (90.4)	181 (79.4)	248 (76.3)	1109 (85.0)	
Histopathological fracture^a^	125 (16.6)	41 (18.0)	52 (16.0)	218 (16.7)	0.824
Leukocytes^a^	56 (7.4)	30 (13.2)	46 (14.2)	132 (10.1)	< 0.001*
Hemoglobin^a^	109 (14.5)	49 (21.5)	105 (32.3)	263 (20.2)	< 0.001*
Alkaline phosphatase^a^	58 (7.7)	17 (7.5)	80 (24.6)	155 (11.9)	< 0.001*
Symptoms and signs^a^					
Redness and hyperemia	7 (0.9)	4 (1.8)	7 (2.2)	18 (1.4)	0.249
Swelling	163 (21.7)	81 (35.5)	143 (44.0)	387 (29.7)	< 0.001*
Warmth	7 (0.9)	5 (2.2)	24 (7.4)	36 (2.8)	< 0.001*
Pain	341 (45.3)	161 (70.6)	241 (74.2)	743 (56.9)	< 0.001*
Dyskinesia	269 (35.8)	106 (46.5)	162 (49.8)	537 (41.1)	< 0.001*
Palpable mass	292 (38.8)	46 (20.2)	126 (38.8)	464 (35.6)	< 0.001*

Data are presented as means ± standard deviations or as counts with percentages in parentheses

^*^p values below 0.05 are considered statistically significant. ^a^The values represent the count of abnormal results for the corresponding indicator. n, number

**Table 2:** The distribution of imaging modalities among patients on the internal/external dataset

Imaging modalities					Internal/External dataset (n)			
X-ray^a^	CT^b^	T1WI^c^	T2WI^c^	T2WI-FS^c^	Benign (604/148)	Intermediate (176/52)	Malignant (263/62)	Total (1043/262)
√	×	×	×	×	162/43	31/21	17/9	210/73
×	√	×	×	×	70/14	8/7	28/2	106/24
×	×	√	√	×	10/0	7/0	12/0	29/0
√	√	×	×	×	129/64	30/8	21/19	180/90
√	×	√	√	×	69/2	31/2	46/2	146/6
×	√	√	√	×	25/0	6/2	27/1	58/3
√	√	√	√	×	139/5	63/7	112/9	314/21
√	×	√	×	√	0/9	0/3	0/8	0/20
×	√	√	×	√	0/1	0/1	0/0	0/2
√	√	√	×	√	0/10	0/1	0/12	0/23

Unless otherwise indicated, data are numbers (n) of patients

*T1WI* T1-weighted image, *T2WI* T2-weighted image, *T2WI-FS* T2-weighted image with fat-suppression

^a^Single view or multiple views of X-ray images are analyzed. ^b^CT includes non-enhanced computed tomography. ^c^T1WI and T2WI are primarily utilized (if available); when T2WI is lacking, T2WI-FS is employed as an alternative

### The performance of models for classifying primary bone tumors

**Fig. 3:**
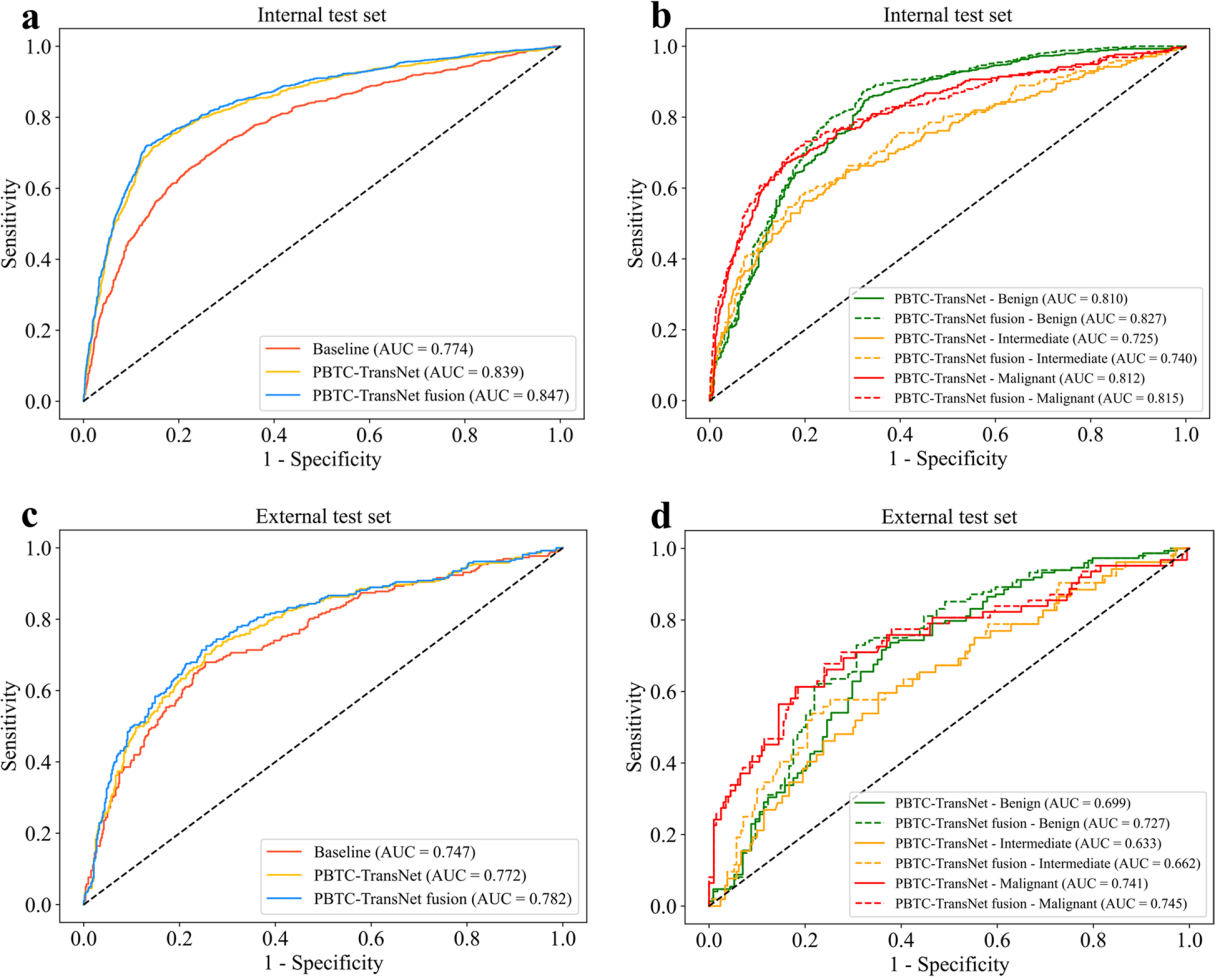
The classification performance of deep learning models on the internal (**a**, **b**) and external (**c**, **d**) test sets. **a**,** c** Receiver operating characteristic (ROC) curves for the Baseline, PBTC-TransNet, and PBTC-TransNet fusion models. **b**, **d** ROC curves for the PBTC-TransNet and PBTC-TransNet fusion models in every class*.* AUC, the area under the ROC; PBTC-TransNet, Primary Bone Tumor Classification Transformer Network

On the internal test set, the PBTC-TransNet performed better than the Baseline model significantly (micro-average AUC: 0.839 vs. 0.774, *p* < 0.001; accuracy: 71.3% vs. 61.4%, *p* < 0.001). While the PBTC-TransNet fusion model slightly improved the performance of the PBTC-TransNet, the difference was not statistically significant, with a micro-average AUC of 0.847 (*p* = 0.362) and an accuracy of 72.5% (*p* = 0.349). Regarding the classification of benign, intermediate, and malignant PBTs, the PBTC-TransNet fusion model achieved AUCs of 0.827 (95% CI: 0.801, 0.852), 0.740 (95% CI: 0.696, 0.784), and 0.815 (95% CI: 0.781, 0.847), respectively (Table 3 and Fig. 3). On the external test set, the PBTC-TransNet achieved a notable micro-average AUC of 0.772 (95% CI: 0.736, 0.807), while the PBTC-TransNet fusion model gained a higher micro-average AUC of 0.782 (95% CI: 0.749, 0.817). For classifying PBTs as benign, intermediate, and malignant, the PBTC-TransNet fusion model achieved AUCs of 0.727 (95% CI: 0.663, 0.791), 0.662 (95% CI: 0.575, 0.748), and 0.745 (95% CI: 0.662, 0.818), respectively, which were higher than those of the PBTC-TransNet (Table 3). Thus, the PBTC-TransNet fusion model was selected for subsequent analysis in this study. In addition, we have shown the importance of clinical characteristics using SHAP for predicting the classification of benign, intermediate, and malignant PBTs, as illustrated in Fig. S3, respectively. The top 3 predictors based on SHAP values for benign PBTs were age, pain, and overall location (Fig. S3a). For intermediate PBTs, the top 3 predictors were age, pain, and palpable mass (Fig. S3b). For malignant PBTs, the top 3 predictors were age, alkaline phosphatase, and pain (Fig. S3c). Of the significant predictors identified, redness and hyperemia were the least important predictors in all tumor classifications. We have included additional performance metrics including recall and F1 score in Table S5 and Table S6, providing a comprehensive overview of the models' performance.

**Table 3:** Performance of deep learning models for primary bone tumor classification on the internal and external test sets

Models	Internal test set				External test set			
	AUC	Accuracy	Sensitivity	Specificity	AUC	Accuracy	Sensitivity	Specificity
Baseline^a^	0.774 (0.756, 0.791)	61.4 (58.4, 64.2)	61.4 (58.2, 64.3)	80.7 (78.9, 82.3)	0.747 (0.708, 0.783)	61.1 (55.3, 66.8)	61.1 (55.3, 66.8)	80.5 (77.0, 83.9)
PBTC-TransNet^a^	0.839 (0.823, 0.854)	71.3 (68.6, 74.0)	71.3 (68.3, 74.0)	85.7 (84.2, 87.1)	0.772 (0.736, 0.807)	61.5 (55.3, 67.6)	61.5 (55.3, 67.2)	80.7 (77.2, 84.1)
Benign	0.810 (0.782, 0.838)	77.2 (74.5, 79.8)	75.1 (72.5, 77.8)	62.2 (57.7, 67.0)	0.699 (0.632, 0.762)	66.8 (60.7, 72.9)	63.7 (58.2, 69.0)	39.5 (30.4, 48.7)
Intermediate	0.725 (0.679, 0.765)	84.1 (82.0, 86.2)	64.2 (60.6, 67.8)	94.1 (92.4, 95.7)	0.633 (0.549, 0.715)	77.1 (71.8, 82.1)	52.4 (47.8, 57.4)	93.3 (89.9, 96.6)
Malignant	0.812 (0.779, 0.844)	81.4 (78.9, 83.6)	73.5 (70.0, 76.6)	89.3 (87.1, 91.5)	0.741 (0.657, 0.820)	79.0 (74.0, 84.0)	65.7 (59.0, 72.1)	91.0 (86.7, 94.8)
PBTC-TransNet fusion^a^	0.847 (0.832, 0.862)	72.5 (69.8, 75.2)	72.5 (69.6, 75.2)	86.2 (84.7, 87.7)	0.782 (0.749, 0.817)	63.0 (56.9, 68.7)	63.0 (57.3, 68.7)	81.5 (78.0, 84.8)
Benign	0.827 (0.801, 0.852)	79.0 (76.6, 81.6)	77.0 (74.4, 79.6)	64.8 (60.4, 69.3)	0.727 (0.663, 0.791)	68.7 (63.0, 74.4)	65.6 (60.1, 70.9)	42.1 (33.0, 51.7)
Intermediate	0.740 (0.696, 0.784)	83.3 (81.1, 85.6)	66.5 (62.8, 70.2)	91.8 (90.0, 93.6)	0.662 (0.575, 0.748)	78.2 (72.9, 83.2)	53.9 (49.1, 59.4)	94.3 (91.0, 97.4)
Malignant	0.815 (0.781, 0.847)	82.6 (80.4, 84.9)	73.3 (70.2, 76.3)	92.1 (90.0, 93.9)	0.745 (0.662, 0.818)	79.0 (74.0, 84.0)	66.2 (59.6, 72.7)	90.5 (86.1, 94.3)
*p* value (Baseline vs. PBTC-TransNet)	< 0.001*	< 0.001*	/	/	0.462	1.000	/	/
*p* value (PBTC-TransNet vs. PBTC-TransNet fusion)	0.362	0.349	/	/	0.609	0.046*	/	/

Accuracy, sensitivity, and specificity are expressed as percentages. Data in parentheses are 95% confidence intervals

*AUC* the area under the receiver operating characteristic curve, *PBTC-TransNet* Primary Bone Tumor Classification Transformer Network

^*^p values below 0.05 are considered statistically significant. ^a^The micro-average method is applied to calculate the AUC, sensitivity, and specificity

### Stratification analysis based on imaging modalities

**Fig. 4:**
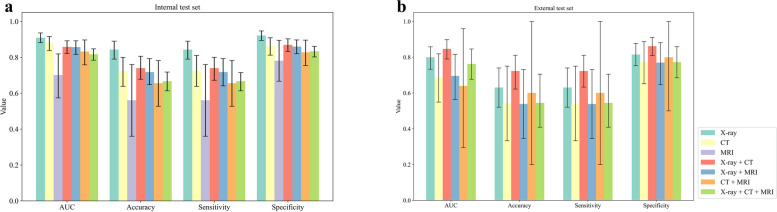
The classification performance of the PBTC-TransNet fusion model in stratification analysis. **a**, **b** Histograms for the performance of the PBTC-TransNet fusion model in different subgroups stratified by the distribution of imaging modalities among patients on the internal and external test sets. Error bars represent the 95% confidence interval of indicators. AUC, the area under the receiver operating characteristic curve; PBTC-TransNet, Primary Bone Tumor Classification Transformer Network

**Fig. 5:**
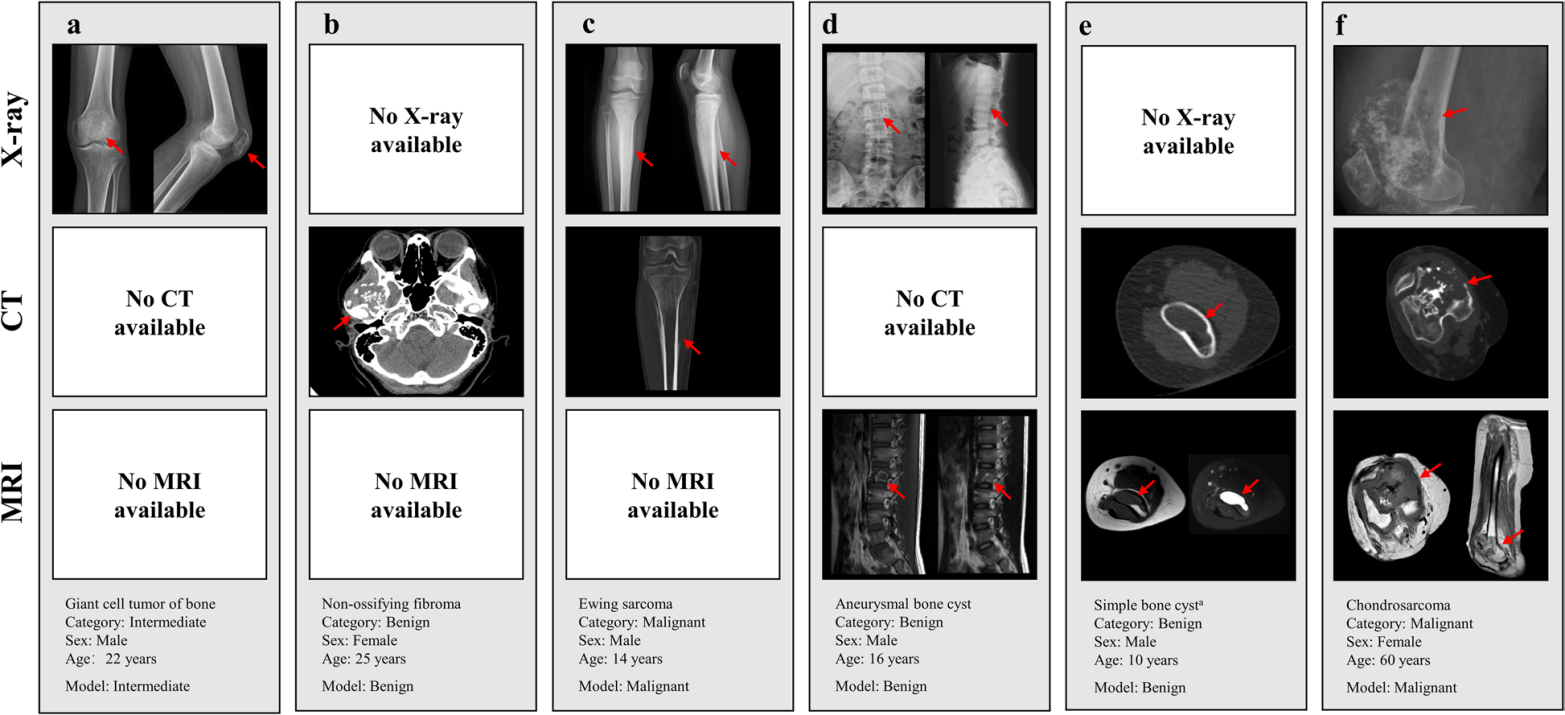
Representative examples (red arrow) correctly classified by the PBTC-TransNet fusion model on the external test set. ^a^T2-weighted image (T2WI) with fat-suppression is provided to the model when T2WI was not available. PBTC-TransNet, Primary Bone Tumor Classification Transformer Network

**Fig. 6:**
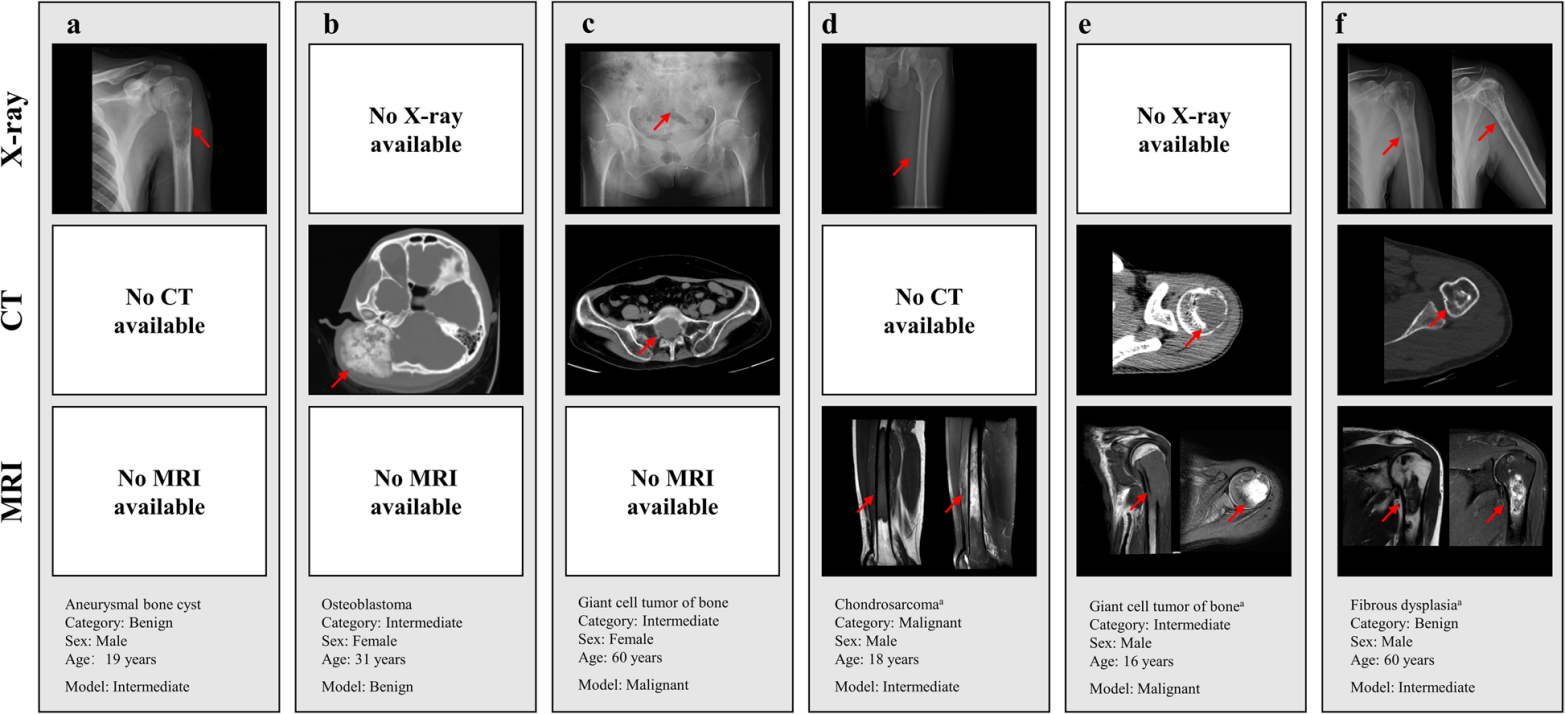
Representative examples (red arrow) incorrectly classified by the PBTC-TransNet fusion model on the external test set. ^a^T2-weighted image (T2WI) with fat-suppression is provided to the model when T2WI was not available. PBTC-TransNet, Primary Bone Tumor Classification Transformer Network

The PBTC-TransNet fusion model displayed varying classification performance across different patient subgroups stratified by the distribution of imaging modalities. On the internal test set, the PBTC-TransNet fusion model gained micro-average AUCs ranging from 0.700 to 0.909 and accuracies ranging from 56.0% to 84.3% across all patient subgroups (Table 4 and Fig. 4a). Notably, it showed the highest micro-average AUC of 0.909, accuracy of 84.3%, micro-average sensitivity of 84.3%, and micro-average specificity of 92.1% in those with only X-rays. For patient subgroups with X + CT and those with X-ray + CT + MRI, the model effectively classified them with a micro-average AUC of 0.858 and 0.816, respectively. On the external test set, the PBTC-TransNet fusion model achieved the highest micro-average AUC of 0.847 for patients with X-ray + CT. For all subgroups, the model showed micro-average AUCs of 0.640 to 0.847 and accuracies of 53.8% to 72.2%, with wide 95% CIs (Fig. 4b). Representative examples correctly and incorrectly classified by the PBTC-TransNet fusion model on the external test set are shown in Figs. 5 and 6. It was worth noting that the external dataset did not include patients with only MRI examination (Table 2). The performance of the PBTC-TransNet fusion model was further analyzed by stratifying the patient data based on age, gender, and time period. The results, presented in Tables S7, S8, and S9, indicate variations in model performance across different demographic groups.

**Table 4:** The classification performance of the PBTC-TransNet fusion model stratified by the distribution of imaging modalities among patients

Imaging modalities	Internal test set				External test set			
	AUC^a^	Accuracy	Sensitivity^a^	Specificity^a^	AUC^a^	Accuracy	Sensitivity^a^	Specificity^a^
X-ray	0.909 (0.882, 0.936)	84.3 (79.0, 89.0)	84.3 (79.0, 89.0)	92.1 (89.4, 94.7)	0.799 (0.733, 0.858)	63.0 (52.1, 74.0)	63.0 (52.1, 74.0)	81.5 (75.3, 87.7)
CT	0.880 (0.837, 0.916)	72.4 (63.8, 80.0)	72.4 (63.8, 81.0)	86.2 (81.2, 90.9)	0.687 (0.549, 0.819)	54.2 (33.3, 75.0)	54.2 (33.3, 75.0)	77.1 (65.2, 88.7)
MRI	0.700 (0.574, 0.819)	56.0 (36.0, 76.0)	56.0 (36.0, 76.0)	78.0 (66.7, 89.5)	NA	NA	NA	NA
X-ray + CT	0.858 (0.822, 0.892)	73.9 (67.8, 80.6)	73.9 (67.2, 80.0)	86.9 (83.3, 90.3)	0.847 (0.790, 0.898)	72.2 (62.2, 81.1)	72.2 (63.3, 81.1)	86.1 (81.0, 91.0)
X-ray + MRI	0.856 (0.816, 0.892)	71.7 (64.8, 79.3)	71.7 (64.1, 79.3)	85.9 (82.0, 89.7)	0.695 (0.564, 0.816)	53.8 (34.6, 73.1)	53.8 (34.6, 73.1)	76.9 (64.6, 88.2)
CT + MRI	0.831 (0.758, 0.897)	65.5 (52.7, 78.2)	65.5 (52.7, 78.2)	82.7 (75.4, 89.6)	0.640 (0.295, 0.960)	60.0 (20.0, 100.0)	60.0 (20.0, 100.0)	80.0 (50.0, 100.0)
X-ray + CT + MRI	0.816 (0.784, 0.847)	66.6 (61.3, 71.8)	66.6 (61.3, 71.5)	83.3 (80.2, 86.1)	0.762 (0.677, 0.846)	54.5 (40.9, 70.5)	54.5 (40.9, 70.5)	77.3 (68.5, 85.9)

Accuracy, sensitivity, and specificity are expressed as percentages. Data in parentheses are 95% confidence intervals

*PBTC-TransNet* Primary Bone Tumor Classification Transformer Network, *AUC* the area under the receiver operating characteristic curve, *NA* not available

^a^The micro-average method is applied to calculate the AUC, sensitivity, and specificity

## Discussion

In this study, we developed the PBTC-TransNet fusion model, which leverages incomplete multimodal images from X-ray, CT, and MRI, along with clinical characteristics, to accurately classify PBTs as benign, intermediate, or malignant. The model demonstrated good performance, achieving a micro-average AUC of 0.847 on the internal test set and a micro-average AUC of 0.782 on the external test set.

Accurate classification of PBTs is essential to ensure their effective treatment and management [3]. Previous studies have built single-modal models based on EfficientNet or Mask-RCNN-X101 to classify PBTs, achieving an AUC of 0.79 and an accuracy of 80.2% on the external test set, respectively [11, 12]. Our team proposed an ensemble multi-task framework that simultaneously detects, segments, and classifies PBTs and bone infections and subclassifies the benign, intermediate, and malignant PBTs on MRI [13]. While these approaches represent significant advancements, they are primarily limited to single-modal data, and their clinical applicability diminishes when faced with the common issue of incomplete multimodal images in real-world settings [11–13, 25]. In contrast, our current study addresses this critical gap by developing the PBTC-TransNet fusion model, which is specifically designed to handle incomplete multimodal images from X-ray, CT, and MRI, alongside clinical characteristics. This design enables our model to maintain robust performance even when certain imaging modalities are unavailable, thereby enhancing its clinical relevance and applicability in diverse healthcare environments. We selected EfficientNet, which is recognized as a state-of-the-art (SOTA) model for classification tasks, as the baseline model for quantitative comparison. This choice was made due to EfficientNet’s proven effectiveness and efficiency in medical image classification [12, 26, 27]. By comparing our PBTC-TransNet fusion model with this baseline, we demonstrated that our approach not only matches or exceeds the performance of these widely used models but also effectively handles cases where certain imaging modalities are missing. Machine learning techniques to discriminate bone lesions have achieved relatively good performance across various imaging modalities, with reported AUC values ranging from 0.73 to 0.96 in several cohort studies [28]. However, most studies that have developed machine learning classification models for bone tumors are preliminary and limited by small sample sizes and retrospective analyses [28]. To our knowledge, there are currently no studies specifically focused on bone tumor classification models that utilize multimodal fusion methods. Nevertheless, some studies highlight the promise of multimodal techniques, which can integrate data from different imaging modalities along with clinical information, offering significant potential for improving diagnostic accuracy and comprehensiveness [29, 30]. In this study, we developed the PBTC-TransNet fusion model to fully utilize patients’ incomplete multimodal images and clinical characteristics, which is expected to be applied to classify a broader PBT population and better fit in real clinical scenarios.

Strategies for handling the samples with missing modalities include direct discarding, data imputation techniques, and separate model training based on the available data for each modality [15, 31, 32]. Nevertheless, these strategies have limitations, such as ignoring valuable information, introducing unnecessary noise, or failing to exploit the correlations across multiple modalities, which potentially compromises classification performance [15, 16]. To effectively integrate information from different image modalities, we deployed the Transformer networks and Bernoulli indicators in our study. The Bernoulli indicators were used for every modality to simulate real-world scenarios where multimodal images might be incomplete [33]. This allowed our model to adapt to situations where certain imaging modalities are unavailable. The Transformer took advantage of the attention mechanism to foster the establishment of long-range dependencies both within and across distinct imaging modalities, facilitating the efficient amalgamation of information sourced from multiple modalities [17]. Moreover, to address missing clinical characteristics, we implemented an iterative imputation strategy and then integrated it into the PBTC-TransNet fusion model to simulate the actual diagnostic process of radiologists. These design strategies enhanced the model’s ability to account for incomplete clinical information, thereby improving its overall performance and clinical relevance. By effectively addressing missing clinical characteristics and mimicking real-world diagnostic scenarios, our model demonstrates promising potential for widespread adoption and generalizability in diverse clinical settings. The SHAP analysis highlights that age, pain, and overall location are crucial for distinguishing benign, intermediate, and malignant PBTs. These insights from the SHAP analysis suggest that focusing on these clinical characteristics in practice could enhance diagnostic accuracy and improve patient outcomes. Integrating such detailed impact analyses into predictive models can provide more robust and clinically relevant tools for radiologists.

Accurate diagnosis and timely treatment are crucial for patients with malignant PBTs to prevent their progressions and potentially life-threatening complications [1]. Our PBTC-TransNet fusion model demonstrated strong performance in identifying malignant PBTs, achieving high accuracies of 82.6% on the internal test set and 79.0% on the external test set. Visualization analysis revealed that our model effectively recognized characteristic imaging manifestations of malignant PBTs, such as tumor bone, soft‑tissue mass, and invasive periosteal reaction (Fig. 5c and f). Moreover, we observed that most patients with malignant PBTs have complete multimodal images on the internal (112 of 263 patients) and external (21 of 62 patients) test sets, which provided valuable information and clues for classification. Unfortunately, our model occasionally failed to diagnose tumors with atypical imaging appearances, such as those lacking evident bone destruction (Fig. 6d). Future research efforts should focus on enhancing the model’s ability to recognize such atypical imaging cases, thereby further improving classification accuracy and clinical utility.

Accurately and timely diagnosis of benign PBTs based on medical images is significant for avoiding unnecessary expensive and invasive examinations [34]. The PBTC-TransNet fusion model displayed satisfactory classification performance with an AUC of 0.827 for benign PBTs on the internal test set. Many benign PBTs with typical imaging manifestations are easily identified (Fig. 5b). For example, among the 195 osteochondromas included in our study, which typically present as bony protuberances with well-defined boundaries extending into soft tissue, the fusion model correctly identified 180 cases [1]. This highlights the model's ability to accurately recognize benign PBTs with typical imaging manifestations, thereby facilitating their timely diagnosis and appropriate management.

Classifying intermediate PBTs before surgery poses a significant challenge due to their potential to present both benign and malignant imaging features [1]. Previous studies have attempted binary classification models based on radiographs and MRI for differentiating benign and malignant bone lesions [11, 12], but these models are not sufficient for intermediate PBTs, as they cannot be simply categorized as benign PBTs and often demand subsequent interventions beyond those typically prescribed for benign lesions [1, 35]. Another study proposed a triple classification model that incorporated patient clinical characteristics and radiographs, achieving high accuracy (85.1%) in classifying intermediate PBTs [36]. In comparison, our PBTC-TransNet fusion model, which is based on incomplete multimodal imaging, showed comparable performance, with an accuracy of 83.3% on the internal test set. Remarkably, even when applied to the external test set, which mainly included patients who underwent only X-ray imaging (40.4%), limiting the available information for classification (Table S4), the PBTC-TransNet fusion model achieved an accuracy of 78.2%. This result underscores the robust generalization capabilities of the PBTC-TransNet fusion model across different datasets and imaging modalities.

A model that robustly deals with incomplete data subsets from various modalities demonstrates strong applicability in real-world clinical settings [17]. The model exhibited the best classification results (micro-average AUC: 0.909) within the patient subgroup solely reliant on X-rays on the internal test set, despite the inherent limitations of X-rays, such as superimpositions and soft tissue resolution [1]. This success was largely attributed to most of them (90 of 210 patients) having osteochondroma, which with typically identified imaging manifestations. However, the heterogeneous and rare PBTs are difficult to classify and often require further MRI examination in clinical practice [37]. For patient subgroups that underwent MRI examinations (MRI, X + MRI, CT + MRI, and X + CT + MRI) on the internal test set, the PBTC-TransNet fusion model gained slightly inferior classification performance. It was interpreted that more patients in these subgroups suffered difficult-to-classify rare PBTs, such as lymphoma and haemangioma (Table S4) [38, 39]. On the external test set, the stratification analysis results of the model were less significant, with wide 95% CIs for several patient subgroups (CT, X + MRI, and CT + MRI), primarily due to the limited sample size (Table 2). In the future, prospective multicenter studies with larger datasets are needed to validate the model’s classification performance in real-world clinical practice settings. Other imaging techniques can also provide additional information for the classification of bone tumors [7]. For example, Diffusion-weighted imaging has been proven to provide valuable information for characterizing benign and malignant musculoskeletal tumors [40]. Considering the inclusion of this imaging sequence in future studies may further improve the accuracy of tumor classification. The stratified analysis revealed that the PBTC-TransNet fusion model performed optimally in the 11–19 years age group and among female patients, while performance was comparatively lower in older age groups and among male patients. These findings underscore the importance of considering demographic factors in model development and highlight areas for future optimization.

Our study had several limitations. First, there may be potential selection bias because we only retrospectively studied histopathologically confirmed cases of PBTs, excluding clinically diagnosed cases. Second, despite the proven value of dynamic contrast-enhanced images in PBT diagnosis, we did not incorporate them into our model development. This decision was influenced by factors such as the variability in patient compliance with contrast-enhanced imaging, as well as concerns regarding risks associated with gadolinium deposition and patient anxiety [41–43]. Third, we did not perform visualization for our models, which might limit their clinical application due to the inherent “black-box” nature of DL techniques. Future work will focus on implementing visualization methods, such as Grad-CAM, to interpret and gain deeper insights into the predictions made by our model. In addition, we have yet to quantify the impact of different modality combinations on the model’s performance. Subsequent research will aim to assess the clinical benefits of our model across different modality combinations. Fourth, the external test set was relatively small, with only 262 patients, which may limit the robustness of the validation. While the model's consistent performance across datasets provides some reassurance, we plan to include larger and more diverse external datasets in future studies to further validate the model's generalizability. Finally, due to the retrospective nature and data limitations of our study, we were unable to include patient outcomes and follow-up data. Future prospective studies will address this by collecting detailed patient outcomes to better assess the long-term impact and clinical utility of the PBTC-TransNet model.

## Conclusion

In conclusion, we successfully developed and externally validated the PBTC-Transnet fusion model, using incomplete multimodal images from X-ray, CT, and MRI, along with clinical characteristics to effectively classify PBTs into benign, intermediate, or malignant. The model aptly mirrors real-life clinical scenarios, which is expected to provide guidance for clinical decision-making and potentially improve patient outcomes. Moreover, our proposed model holds promise for application in other diseases characterized by incomplete multimodal images, promising fresh insights into the realm of computer-aided diagnosis of medical images.

## Supplementary Information

Supplementary Material 1.

Supplementary Material 2.

## Data Availability

No datasets were generated or analysed during the current study.

## Abbreviations

AUC: The areas under the receiver operating characteristic curve
CI: Confidence interval
CLAIM: Checklist for Artificial Intelligence in Medical Imaging
CT: Computed tomography
DICOM: Digital Imaging and Communications in Medicine
DL: Deep learning
GDPR: General Data Protection Regulation
HIPAA: Health Insurance Portability and Accountability Act
IoU: Intersection over union
MRI: Magnetic resonance imaging
NCCN: National Comprehensive Cancer Network
PBT: Primary bone tumor
PBTC-TransNet: Primary Bone Tumor Classification Transformer Network
RROI: Rectangular region of interest
SHAP: Shapley Additive Explanations
T1WI: T1-weighted image
T2WI: T2-weighted image
T2WI-FS: T2-weighted image with fat-suppression
WHO: World Health Organization
