# Supplementary material for: A deep learning model to enhance the classification of primary bone tumors based on incomplete multimodal images in X-ray, CT, and MRI
Source: Cancer Imaging. 2024 Oct 10;24:135. doi: 10.1186/s40644-024-00784-7 (PMC11468403; doi:10.1186/s40644-024-00784-7)
Supplement: Supplementary file 1 — Supplementary Material 1. [file 40644_2024_784_MOESM1_ESM.docx]

**Supplementary Materials 1**

**Appendix S1** Stratification of the internal dataset into training, validation, and internal test sets

**Appendix S2** Detailed information about X-rays

**Appendix S3** Deep learning model development

**Appendix S4** Imputation for missing clinical data

**Appendix S5** Model evaluation

**Table S1** A one-fold example of the number of patients selected for different sets in each stratum

**Table S2** Clinical characteristics of patients in the study stratified by datasets

**Table S3** The subtypes and numbers of benign, intermediate, and malignant primary bone tumors on the internal/external dataset

**Table S4** The distribution of imaging modalities among patients with each type of primary bone tumors on the internal/external dataset

**Table S5** Additional performance metrics of deep learning models for primary bone tumor classification on the internal and external test sets

**Table S6** Additional performance metrics of the PBTC-TransNet fusion model stratified by the distribution of imaging modalities among patients

**Table S7** Performance of the PBTC-TransNet fusion model for primary bone tumor classification stratified by age

**Table S8** Performance of the PBTC-TransNet fusion model for primary bone tumor classification stratified by sex

**Table S9** Performance of the PBTC-TransNet fusion model for primary bone tumor classification stratified by time period

**Fig. S1** The architecture of the encoders

**Fig. S2** The variable distribution of each imputed clinical data compared to the original data on the internal (**a**) and external (**b**) datasets

**Fig. S3** SHAP violin plots showing the impact of clinical characteristics on the model's predictions for classifying benign (**a**), intermediate (**b**), and malignant (**c**) PBTs on the external test set. Each violin plot displays the top clinical characteristics based on their SHAP values, which represent the characteristic's contribution to the model's output. The color bar indicates the feature value, with red representing high feature values and blue representing low feature values. SHAP, Shapley Additive Explanations; PBTs, Primary bone tumors

**Appendix S1** Stratification of the internal dataset into training, validation, and internal test sets

This study used incomplete multimodal images from X-ray, CT, and MRI alongside clinical characteristics to classify primary bone tumors (PBTs). To balance the number of patients with different imaging modalities, we performed the stratified random sampling method with five-fold cross-validation to divide the internal dataset into training, validation, and internal test sets. Specifically, all patients were first divided into different strata according to the imaging modalities they have, and then patients were randomly selected from each stratum by a ratio of 7:1:2. Next, the selected patients from each stratum were combined to obtain the training (70%), validation (10%), and internal test sets (20%) for the overall internal dataset, respectively. During the five-fold cross-validation, the above selection was repeated five times, where each selected internal test set was ensured to have no patients overlapping with each other. Table S1 shows a one-fold example of the number of patients selected for different sets in each stratum. A separate model was trained for each fold, and predictions were obtained for the internal test set. Finally, predictions of the five-fold internal test sets were aggregated to obtain the overall performance of models on the internal test set.

**Appendix S2** Detailed information about X-rays

If available, multiple views of X-ray images of PBT patients were analyzed, including standard anteroposterior/posteroanterior and lateral views, along with the axial view of the patella and calcaneus. Patients with multiple osteochondromas had multiple X-ray images of different body parts. In center 1, six hundred and forty-four patients underwent X-ray examinations with two views, one hundred and forty-one with one view, and sixty-five with three to ten views. Specifically, six patients had axial and lateral views of the calcaneus. Six patients had axial, anteroposterior/posteroanterior, and lateral views of the patella. Thirty-three patients with multiple osteochondromas had three to ten X-ray images of different body parts. In center 2, one hundred and ninety-five patients underwent X-ray examinations with two views, twenty-two with one view, and sixteen with three to six views. Specifically, four patients had axial and lateral views of the calcaneus. Five patients with multiple osteochondromas had four to six X-ray images of different body parts.

**Appendix S3** Deep learning model development

All images were normalized by a z-score method. Data augmentation techniques were employed during the training phase, consisting of random cropping, rotations (from -30° to 30°), scaling, shear, affine transformation, and flipping from the MONAI library [1]. The model was optimized using the Adam optimizer with a batch size of 12, an initial learning rate of $1\times{10}^{-4}$, and was trained for 500 epochs. The Focal Loss was used as the training objective of models due to the problem of imbalanced data categories [2]. During the test phase of the baseline model, the appropriate model was selected based on the modality of the data, and the output results of each imaging modality-specific model were combined through an average ensemble method.

**Appendix S4** Imputation for missing clinical data

In this study, thirty (2.3%) patients had missing clinical data for patient symptoms and signs, eighty-three (6.4%) for leukocytes, one hundred and seventy-one (13.1%) for hemoglobin, and two hundred and thirty-six (18.1%) for alkaline phosphatase. Various imputation methods are available, including mean, k-nearest neighbors, regression, Bayesian estimation, etc. We used the iterative imputation strategy based on the chained equation forest (Mice-Forest) to impute the missing clinical data for patients. This approach can handle various types of input data and makes minimal assumptions about the data's structure. Regarding the iterative process, we first assumed that there are three missing variables (A, B, and C) in the dataset. We used the average of the initial data of each variable to fill in the missing clinical data. Then, the filling value in variable A was considered missing, and A was regressed on other variables, with A as the dependent variable and the other variables as independent variables. Finally, we used the prediction of the regression curve to estimate the missing data. Similarly, variables A (including estimated values) and C were used to estimate variable B. Iteration was the sequential filling of all missing variables in the dataset. After one iteration, all missing values were replaced by regression-predicted values [3]. In this study, we examined the results of 5 iterations. We present a visual comparison of the filled and initial data distribution for each iteration in Fig. S2. During the filling process, keep the initial values of all variables unchanged and use appropriate predicted values to fill in missing clinical features. The variables of categorical clinical characteristics were imputed directly. For continuous clinical characteristics, specific values were imputed first and then organized into categorical variable labels based on the clinical reference range for that clinical indicator. To ensure data reliability, we refrained from performing imputation for clinical data with a larger proportion of missing values, such as erythrocyte sedimentation rate.

**Appendix S5** Model evaluation

Based on imbalanced datasets on the internal (benign, n = 604; intermediate, n = 176; malignant, n = 263) and external (benign, n = 148; intermediate, n = 52; malignant, n = 62) datasets, the micro-average method was applied to calculate the areas under the receiver operating characteristic curve (AUC), sensitivity, and specificity for evaluation. The calculation formula of the micro-average is:

$\text{Micro-average}=\frac{\sum_{i=1}^{N} \text{T}\text{P}_{i}}{\sum_{i=1}^{N} (\text{T}\text{P}_{i}+\text{F}\text{N}_{i})}\times\frac{\sum_{i=1}^{N} \text{T}\text{N}_{i}}{\sum_{i=1}^{N} (\text{T}\text{N}_{i}+\text{F}\text{P}_{i})}$,

where $N$ is the number of classes, ${TP}_{i}$ is the number of true positives for class $i$, ${TN}_{i}$ is the number of true negatives for class $i$, ${FP}_{i}$ is the number of false positives for class $i$, ${FN}_{i}$ is the number of false negatives for class $i$.

**Reference**

1. Cardoso MJ, Li W, Brown R, Ma N, Kerfoot E, Wang Y, et al. MONAI: An open-source framework for deep learning in healthcare. 2022.

2. Lin T-Y, Goyal P, Girshick R, He K, Dollár P: Focal loss for dense object detection. In: Proceedings of the IEEE international conference on computer vision. 2017: 2980-8.

3. Laatifi M, Douzi S, Ezzine H, Asry CE, Naya A, Bouklouze A, et al. Explanatory predictive model for COVID-19 severity risk employing machine learning, shapley addition, and LIME. Scientific reports. 2023;13(1):5481; doi: 10.1038/s41598-023-31542-7.

**Table S1** A one-fold example of the number of patients selected for different sets in each stratum

| **Imaging modalities** | **Training (n = 730)** | **Validation (n = 104)** | **Internal test (n = 209)** |
| --- | --- | --- | --- |
| X-ray | 147 | 21 | 42 |
| CT | 74 | 11 | 21 |
| MRI | 20 | 3 | 6 |
| X-ray + CT | 126 | 18 | 36 |
| X-ray + MRI | 102 | 15 | 29 |
| CT + MRI | 41 | 5 | 12 |
| X-ray + CT + MRI | 220 | 31 | 63 |

n, number

**Table S2** Clinical characteristics of patients in the study stratified by datasets

| **Characteristic** | **Internal**  **(n = 1043)** | **External**  **(n = 262)** | **All**  **(n = 1305)** | ***p* value** |
| --- | --- | --- | --- | --- |
| Age (years) | 24 ± 18 | 30 ± 19 | 26 ± 18 | ＜0.001* |
| Sex: female | 411 (39.4) | 101 (38.5) | 512 (39.2) | 0.800 |
| Locations overall |  |  |  | 0.039* |
| Torso or head | 146 (14) | 50 (19.1) | 196 (15.0) |  |
| Extremities | 897 (86) | 212 (80.9) | 1109 (85.0) |  |
| Histopathological fracture^a^ | 159 (15.2) | 59 (22.5) | 218 (16.7) | 0.005* |
| Leukocytes^a^ | 88 (8.4) | 44 (16.8) | 132 (10.1) | ＜0.001* |
| Hemoglobin^a^ | 208 (19.9) | 55 (21) | 263 (20.2) | 0.705 |
| Alkaline phosphatase^a^ | 150 (14.4) | 5 (1.9) | 155 (11.9) | ＜0.001* |
| Symptoms and signs^a^ |  |  |  |  |
| Redness and hyperemia | 11 (1.1) | 7 (2.7) | 18 (1.4) | 0.045* |
| Swelling | 301 (28.9) | 86 (32.8) | 387 (29.7) | 0.209 |
| Warmth | 13 (1.2) | 23 (8.8) | 36 (2.8) | ＜0.001* |
| Pain | 600 (57.5) | 143 (54.6) | 743 (56.9) | 0.389 |
| Dyskinesia | 440 (42.2) | 97 (37) | 537 (41.1) | 0.129 |
| Palpable mass | 372 (35.7) | 92 (35.1) | 464 (35.6) | 0.868 |
| Tumor distribution |  |  |  | 0.513 |
| Benign | 604 (57.9) | 148 (56.5) | 752 (57.6) |  |
| Intermediate | 176 (16.9) | 52 (19.8) | 228 (17.5) |  |
| Malignant | 263 (25.2) | 62 (23.7) | 325 (24.9) |  |

Data are presented as means ± standard deviations or as counts with percentages in parentheses. **p* values below 0.05 are considered statistically significant. ^a^The values represent the count of abnormal results for the corresponding indicator. n, number

**Table S3** The subtypes and numbers of benign, intermediate, and malignant primary bone tumors on the internal/external dataset

| **Benign** | **Internal/External  (n = 604/148)** |  | **Intermediate** | **Internal/External  (n = 176/52)** |  | **Malignant** | **Internal/External  (n = 263/62)** |
| --- | --- | --- | --- | --- | --- | --- | --- |
| Osteochondroma | 195/32 |  | Giant cell tumor of bone | 79/27 |  | Osteosarcoma | 134/30 |
| Aneurysmal bone cyst | 64/25 |  | Chondromatosis | 46/12 |  | Chondrosarcoma | 37/11 |
| Enchondroma | 68/19 |  | Langerhans cell histiocytosis | 40/11 |  | Ewing sarcoma | 35/7 |
| Simple bone cyst | 55/27 |  | Osteofibrous dysplasia-like Adamantinoma | 6/0 |  | Chordoma | 23/0 |
| Fibrous dysplasia | 55/16 |  | Osteoblastoma | 3/1 |  | Plasmacytoma of bone | 6/11 |
| Osteoid osteoma | 54/9 |  | Desmoplastic fibroma | 1/1 |  | Undifferentiated pleomorphic sarcoma | 5/1 |
| Non-ossifying fibroma | 42/9 |  | Epithelioid haemangioma | 1/0 |  | Leiomyosarcoma | 6/0 |
| Osteofibrous dysplasia | 28/5 |  |  |  |  | Lymphoma | 4/1 |
| Chondroblastoma | 18/1 |  |  |  |  | Epithelioid haemangioendothelioma | 4/0 |
| Osteoma | 9/3 |  |  |  |  | Adamantinoma of long bones | 3/0 |
| Haemangioma | 6/2 |  |  |  |  | Undifferentiated small round cell sarcomas of bone and soft tissue | 3/0 |
| Lipoma | 4/0 |  |  |  |  | Fibrosarcoma | 1/0 |
| Bizarre parosteal osteochondromatous proliferation | 3/0 |  |  |  |  | Angiosarcoma | 1/0 |
| Subungual exostosis | 2/0 |  |  |  |  | Malignant giant cell tumor of bone | 1/0 |
| Chondromyxiod fibroma | 1/0 |  |  |  |  | Malignant non-ossifying fibroma | 0/1 |

n, number

**Table S4** The distribution of imaging modalities among patients with each type of primary bone tumor on the internal/external dataset

| **Primary bone tumor** | **X-ray (n = 210/73)** | **CT  (n = 106/24)** | **MRI  (n = 29/0)** | **X-ray + CT  (n = 180/90)** | **X-ray + MRI  (n = 146/26)** | **CT + MRI  (n = 58/5)** | **X-ray + CT + MRI  (n = 314/44)** | **Total  (n = 1043/262)** |
| --- | --- | --- | --- | --- | --- | --- | --- | --- |
| Osteochondroma | 90/1 | 17/0 | 3/0 | 47/29 | 14/0 | 4/0 | 20/2 | 195/32 |
| Aneurysmal bone cyst | 10/12 | 0/0 | 1/0 | 11/7 | 12/4 | 4/0 | 26/2 | 64/25 |
| Enchondroma | 22/5 | 11/1 | 1/0 | 11/10 | 9/2 | 1/0 | 13/1 | 68/19 |
| Simple bone cyst | 8/14 | 6/3 | 1/0 | 10/3 | 13/3 | 4/1 | 13/3 | 55/27 |
| Fibrous dysplasia | 7/5 | 8/2 | 1/0 | 18/5 | 3/1 | 2/0 | 16/3 | 55/16 |
| Osteoid osteoma | 4/2 | 13/1 | 1/0 | 15/3 | 2/1 | 4/0 | 15/2 | 54/9 |
| Non-ossifying fibroma | 12/4 | 3/2 | 1/0 | 7/2 | 5/0 | 3/0 | 11/1 | 42/9 |
| Osteofibrous dysplasia | 2/0 | 3/4 | 0/0 | 1/0 | 8/0 | 0/0 | 14/1 | 28/5 |
| Chondroblastoma | 4/0 | 1/0 | 1/0 | 5/1 | 0/0 | 1/0 | 6/0 | 18/1 |
| Osteoma | 0/0 | 7/1 | 0/0 | 1/2 | 0/0 | 1/0 | 0/0 | 9/3 |
| Haemangioma | 2/0 | 0/0 | 0/0 | 1/2 | 0/0 | 0/0 | 3/0 | 6/2 |
| Lipoma | 1/0 | 1/0 | 0/0 | 0/0 | 0/0 | 1/0 | 1/0 | 4/0 |
| Bizarre parosteal osteochondromatous proliferation | 0/0 | 0/0 | 0/0 | 1/0 | 1/0 | 0/0 | 1/0 | 3/0 |
| Subungual exostosis | 0/0 | 0/0 | 0/0 | 1/0 | 1/0 | 0/0 | 0/0 | 2/0 |
| Chondromyxiod fibroma | 0/0 | 0/0 | 0/0 | 0/0 | 1/0 | 0/0 | 0/0 | 1/0 |
| Giant cell tumor of bone | 6/13 | 6/3 | 3/0 | 21/3 | 8/4 | 1/1 | 34/3 | 79/27 |
| Chondromatosis | 18/8 | 1/0 | 1/0 | 4/3 | 11/1 | 1/0 | 10/0 | 46/12 |
| Langerhans cell histiocytosis | 6/0 | 0/3 | 2/0 | 4/2 | 9/0 | 2/2 | 17/4 | 40/11 |
| Osteofibrous dysplasia-like Adamantinoma | 1/0 | 0/0 | 1/0 | 0/0 | 3/0 | 0/0 | 1/0 | 6/0 |
| Osteoblastoma | 0/0 | 0/1 | 0/0 | 0/0 | 0/0 | 2/0 | 1/0 | 3/1 |
| Desmoplastic fibroma | 0/0 | 0/0 | 0/0 | 1/0 | 0/0 | 0/0 | 0/1 | 1/1 |
| Epithelioid haemangioma | 0/0 | 1/0 | 0/0 | 0/0 | 0/0 | 0/0 | 0/0 | 1/0 |
| Osteosarcoma | 6/6 | 11/3 | 7/0 | 5/4 | 35/5 | 9/0 | 61/12 | 134/30 |
| Chondrosarcoma | 5/2 | 8/0 | 0/0 | 6/6 | 3/2 | 0/0 | 15/1 | 37/11 |
| Ewing sarcoma | 0/1 | 5/0 | 3/0 | 3/4 | 2/1 | 5/0 | 17/1 | 35/7 |
| Chordoma | 1/0 | 3/0 | 1/0 | 5/0 | 1/0 | 7/0 | 5/0 | 23/0 |
| Plasmacytoma of bone | 1/0 | 0/0 | 0/0 | 0/2 | 0/1 | 3/1 | 2/7 | 6/11 |
| Undifferentiated pleomorphic sarcoma | 0/0 | 0/0 | 1/0 | 0/1 | 0/0 | 2/0 | 2/0 | 5/1 |
| Leiomyosarcoma | 1/0 | 1/0 | 0/0 | 0/0 | 1/0 | 0/0 | 3/0 | 6/0 |
| Lymphoma | 0/0 | 0/0 | 0/0 | 0/1 | 1/0 | 0/0 | 3/0 | 4/1 |
| Epithelioid haemangioendothelioma | 2/0 | 0/0 | 0/0 | 0/0 | 1/0 | 0/0 | 1/0 | 4/0 |
| Adamantinoma of long bones | 1/0 | 0/0 | 0/0 | 1/0 | 1/0 | 0/0 | 0/0 | 3/0 |
| Undifferentiated small round cell sarcomas of bone and soft tissue | 0/0 | 0/0 | 0/0 | 1/0 | 1/0 | 0/0 | 1/0 | 3/0 |
| Fibrosarcoma | 0/0 | 0/0 | 0/0 | 0/0 | 0/0 | 1/0 | 0/0 | 1/0 |
| Angiosarcoma | 0/0 | 0/0 | 0/0 | 0/0 | 0/0 | 0/0 | 1/0 | 1/0 |
| Malignant giant cell tumor of bone | 0/0 | 0/0 | 0/0 | 0/0 | 0/0 | 0/0 | 1/0 | 1/0 |
| Malignant non-ossifying fibroma | 0/0 | 0/0 | 0/0 | 0/0 | 0/1 | 0/0 | 0/0 | 0/1 |

n, number

**Table S5** Additional performance metrics of deep learning models for primary bone tumor classification on the internal and external test sets

| **Models** | **Internal test set** | |  | **External test set** | |
| --- | --- | --- | --- | --- | --- |
|  | **Recall** | **F1 score** |  | **Recall** | **F1 score** |
| Baseline^a^ | 61.4  (58.2, 64.3) | 61.4  (58.4, 64.2) |  | 61.1  (55.3, 66.8) | 61.1  (55.3, 66.8) |
| PBTC-TransNet^a^ | 71.3  (68.3, 74.0) | 71.3  (68.6, 74.0) |  | 61.5  (55.3, 67.2) | 61.5  (55.3, 67.6) |
| Benign | 75.1  (72.5, 77.8) | 77.2  (74.5, 79.8) |  | 63.7  (58.2, 69.0) | 66.8  (60.7, 72.9) |
| Intermediate | 64.2  (60.6, 67.8) | 84.1  (82.0, 86.2) |  | 52.4  (47.8, 57.4) | 77.1  (71.8, 82.1) |
| Malignant | 73.5  (70.0, 76.6) | 81.4  (78.9, 83.6) |  | 65.7  (59.0, 72.1) | 79.0  (74.0, 84.0) |
| PBTC-TransNet fusion^a^ | 72.5  (69.6, 75.2) | 72.5  (69.8, 75.2) |  | 63.0  (57.3, 68.7) | 63.0  (56.9, 68.7) |
| Benign | 77.0  (74.4, 79.6) | 79.0  (76.6, 81.6) |  | 65.6  (60.1, 70.9) | 68.7  (63.0, 74.4) |
| Intermediate | 66.5  (62.8, 70.2) | 83.3  (81.1, 85.6) |  | 53.9  (49.1, 59.4) | 78.2  (72.9, 83.2) |
| Malignant | 73.3  (70.2, 76.3) | 82.6  (80.4, 84.9) |  | 66.2  (59.6, 72.7) | 79.0  (74.0, 84.0) |

Recall and F1 scores are expressed as percentages. Data in parentheses are 95% confidence intervals. ^a^The micro-average method is applied to calculate the Recall and F1 score. PBTC-TransNet, Primary Bone Tumor Classification Transformer Network

**Table S6** Additional performance metrics of the PBTC-TransNet fusion model stratified by the distribution of imaging modalities among patients

| **Imaging modalities** | **Internal test set** | |  | **External test set** | |
| --- | --- | --- | --- | --- | --- |
|  | **Recall** | **F1 score** |  | **Recall** | **F1 score** |
| X-ray | 84.3  (79.0, 89.0) | 84.3  (79.0, 89.0) |  | 63.0  (52.1, 74.0) | 63.0  (52.1, 74.0) |
| CT | 72.4  (63.8, 81.0) | 72.4  (63.8, 80.0) |  | 54.2  (33.3, 75.0) | 54.2  (33.3, 75.0) |
| MRI | 56.0  (36.0, 76.0) | 56.0  (36.0, 76.0) |  | NA | NA |
| X-ray + CT | 73.9  (67.2, 80.0) | 73.9  (67.8, 80.6) |  | 72.2  (63.3, 81.1) | 72.2  (62.2, 81.1) |
| X-ray + MRI | 71.7  (64.1, 79.3) | 71.7  (64.8, 79.3) |  | 53.8  (34.6, 73.1) | 53.8  (34.6, 73.1) |
| CT + MRI | 65.5  (52.7, 78.2) | 65.5  (52.7, 78.2) |  | 60.0  (20.0, 100.0) | 60.0  (20.0, 100.0) |
| X-ray + CT + MRI | 66.6  (61.3, 71.5) | 66.6  (61.3, 71.8) |  | 54.5  (40.9, 70.5) | 54.5  (40.9, 70.5) |

Recall and F1 scores are expressed as percentages. Data in parentheses are 95% confidence intervals. The micro-average method is applied to calculate the Recall and F1 score. PBTC-TransNet, Primary Bone Tumor Classification Transformer Network; NA, not available

**Table S7** Performance of the PBTC-TransNet fusion model for primary bone tumor classification stratified by age

| **Age** | **AUC^a^** | **Accuracy** | **Sensitivity^a^** | **Specificity^a^** | **Recall^a^** | **F1 score^a^** |
| --- | --- | --- | --- | --- | --- | --- |
| Internal test set |  |  |  |  |  |  |
| ≤10 years (n = 271) | 0.866  (0.836, 0.894) | 75.9  (70.7, 80.8) | 75.9  (70.7, 80.8) | 88.0  (85.2, 90.6) | 75.9  (70.7, 80.8) | 75.9  (70.7, 80.8) |
| 11-19 years (n = 255) | 0.925  (0.904, 0.945) | 81.7  (77.0, 86.5) | 81.7  (77.0, 86.5) | 90.9  (88.3, 93.4) | 81.7  (77.0, 86.5) | 81.7  (77.0, 86.5) |
| 20-35 years (n = 263) | 0.807  (0.774, 0.840) | 67.2  (61.7, 72.7) | 67.2  (61.7, 72.7) | 83.6  (80.4, 86.8) | 67.2  (61.7, 72.7) | 67.2  (61.7, 72.7) |
| >35 years (n = 254) | 0.783  (0.746, 0.817) | 64.9  (59.0, 70.9) | 64.9  (59.0, 70.9) | 82.5  (79.0, 85.7) | 64.9  (59.0, 70.9) | 64.9  (59.0, 70.9) |
| External test set |  |  |  |  |  |  |
| ≤14 years (n = 71) | 0.817  (0.744, 0.883) | 76.1  (66.2, 85.9) | 76.1  (66.2, 85.9) | 88.0  (82.2, 93.0) | 76.1  (66.2, 85.9) | 76.1  (66.2, 85.9) |
| 15-25 years (n = 62) | 0.802  (0.731, 0.866) | 67.7  (56.5, 79.0) | 67.7  (56.5, 79.0) | 83.9  (77.2, 90.2) | 67.7  (56.5, 79.0) | 67.7  (56.5, 79.0) |
| 26-46 years (n = 63) | 0.751  (0.674, 0.822) | 56.9  (44.6, 69.2) | 56.9  (44.6, 69.2) | 78.5  (71.2, 85.5) | 56.9  (44.6, 69.2) | 56.9  (44.6, 69.2) |
| >46 years (n = 66) | 0.749  (0.674, 0.827) | 50.0  (37.5, 62.5) | 50.0  (37.5, 62.5) | 75.0  (67.5, 82.3) | 50.0  (37.5, 62.5) | 50.0  (37.5, 62.5) |

Data in parentheses are 95% confidence intervals

^a^ Data are micro-average

*AUC*, the area under the receiver operating characteristic curve *PBTC-TransNet*, Primary Bone Tumor Classification Transformer Network

**Table S8** Performance of the PBTC-TransNet fusion model for primary bone tumor classification stratified by sex

| **Sex** | **AUC^a^** | **Accuracy** | **Sensitivity^a^** | **Specificity^a^** | **Recall^a^** | **F1 score^a^** |
| --- | --- | --- | --- | --- | --- | --- |
| Internal test set |  |  |  |  |  |  |
| Female (n = 411) | 0.855  (0.836, 0.874) | 73.8  (70.2, 77.2) | 73.8  (70.2, 77.2) | 86.9  (85.0, 88.7) | 73.8  (70.2, 77.2) | 73.8  (70.2, 77.2) |
| Male (n = 632) | 0.835  (0.810, 0.860) | 70.5  (66.1, 75.2) | 70.5  (66.1, 75.2) | 85.3  (82.8, 87.7) | 70.5  (66.1, 75.2) | 70.5  (66.1, 75.2) |
| External test set |  |  |  |  |  |  |
| Female (n = 101) | 0.779  (0.718, 0.836) | 62.4  (53.5, 71.3) | 62.4  (53.5, 71.3) | 81.2  (75.9, 86.2) | 62.4  (53.5, 71.3) | 62.4  (53.5, 71.3) |
| Male (n = 161) | 0.783  (0.739, 0.827) | 63.4  (55.9, 70.2) | 63.4  (55.9, 70.2) | 81.7  (77.3, 85.6) | 63.4  (55.9, 70.2) | 63.4  (55.9, 70.2) |

Data in parentheses are 95% confidence intervals

^a^ Data are micro-average

*AUC*, the area under the receiver operating characteristic curve *PBTC-TransNet*, Primary Bone Tumor Classification Transformer Network

**Table S9** Performance of the PBTC-TransNet fusion model for primary bone tumor classification stratified by time period

| **Time period** | **AUC^a^** | **Accuracy** | **Sensitivity^a^** | **Specificity^a^** |
| --- | --- | --- | --- | --- |
| Internal test set |  |  |  |  |
| 2010-2013 (n = 63) | 0.802  (0.731, 0.869) | 70.9  (59.7, 80.6) | 70.9  (59.7, 80.6) | 85.4  (78.9, 91.3) |
| 2014-2017 (n = 346) | 0.862  (0.837, 0.886) | 74.0  (69.3, 78.7) | 74.0  (69.3, 78.7) | 86.9  (84.5, 89.4) |
| 2018-2022 (n = 634) | 0.844  (0.824, 0.863) | 71.8  (68.3, 75.4) | 71.8  (68.3, 75.4) | 85.9  (83.9, 87.8) |
| External test set |  |  |  |  |
| 2010-2013 (n = 40) | 0.676  (0.572, 0.777) | 50.0  (35.0, 67.5) | 50.0  (35.0, 67.5) | 75.0  (65.4, 84.8) |
| 2014-2017 (n = 73) | 0.903  (0.861, 0.945) | 73.9  (63.1, 83.6) | 73.9  (63.1, 83.6) | 86.9  (81.6, 92.3) |
| 2018-2022 (n = 149) | 0.744  (0.694, 0.795) | 61.1  (53.0, 69.2) | 61.1  (53.0, 69.2) | 80.5  (75.9, 84.8) |

Data in parentheses are 95% confidence intervals

^a^ Data are micro-average

*AUC*, the area under the receiver operating characteristic curve *PBTC-TransNet*, Primary Bone Tumor Classification Transformer Network


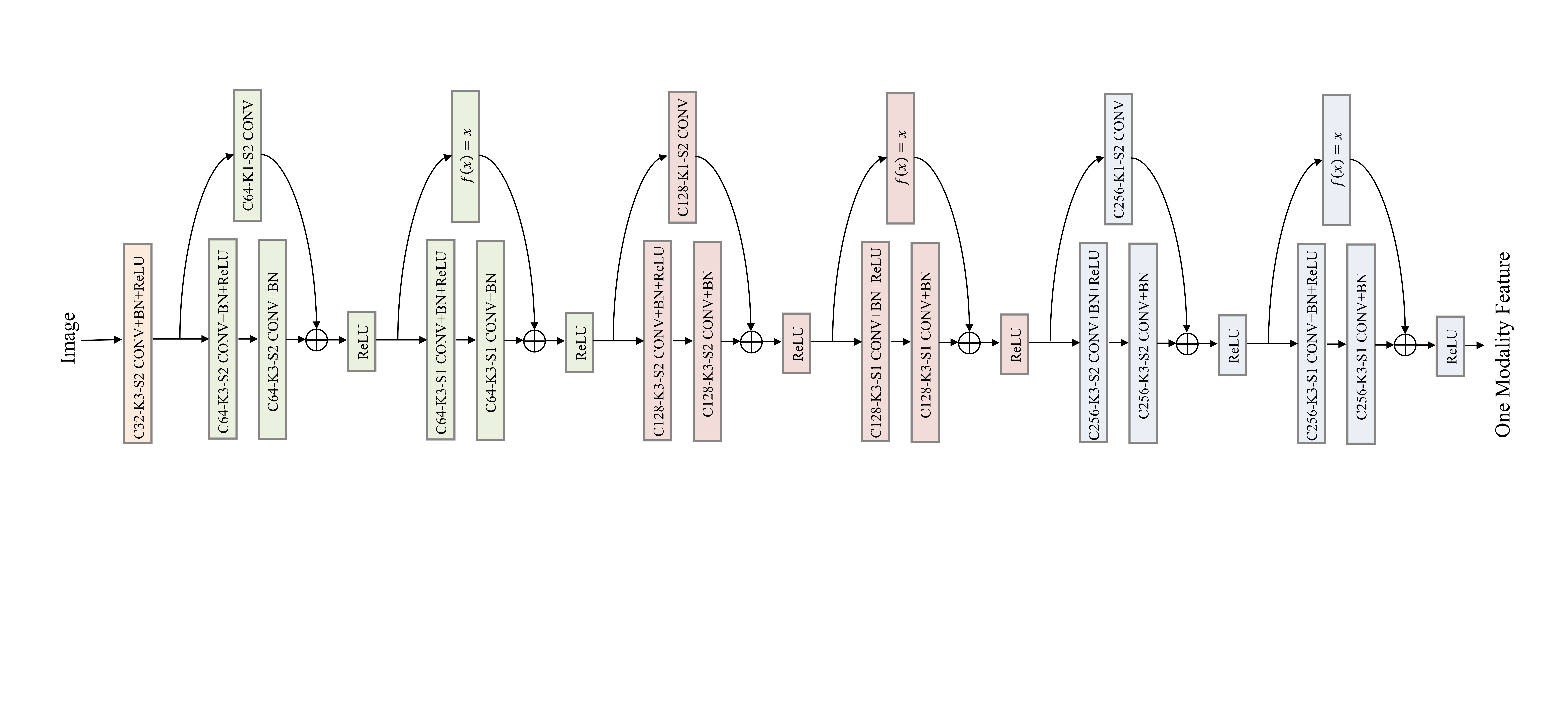


**Fig. S1** The architecture of the encoders. C, Channel; K, Kernel size; S, stride

**
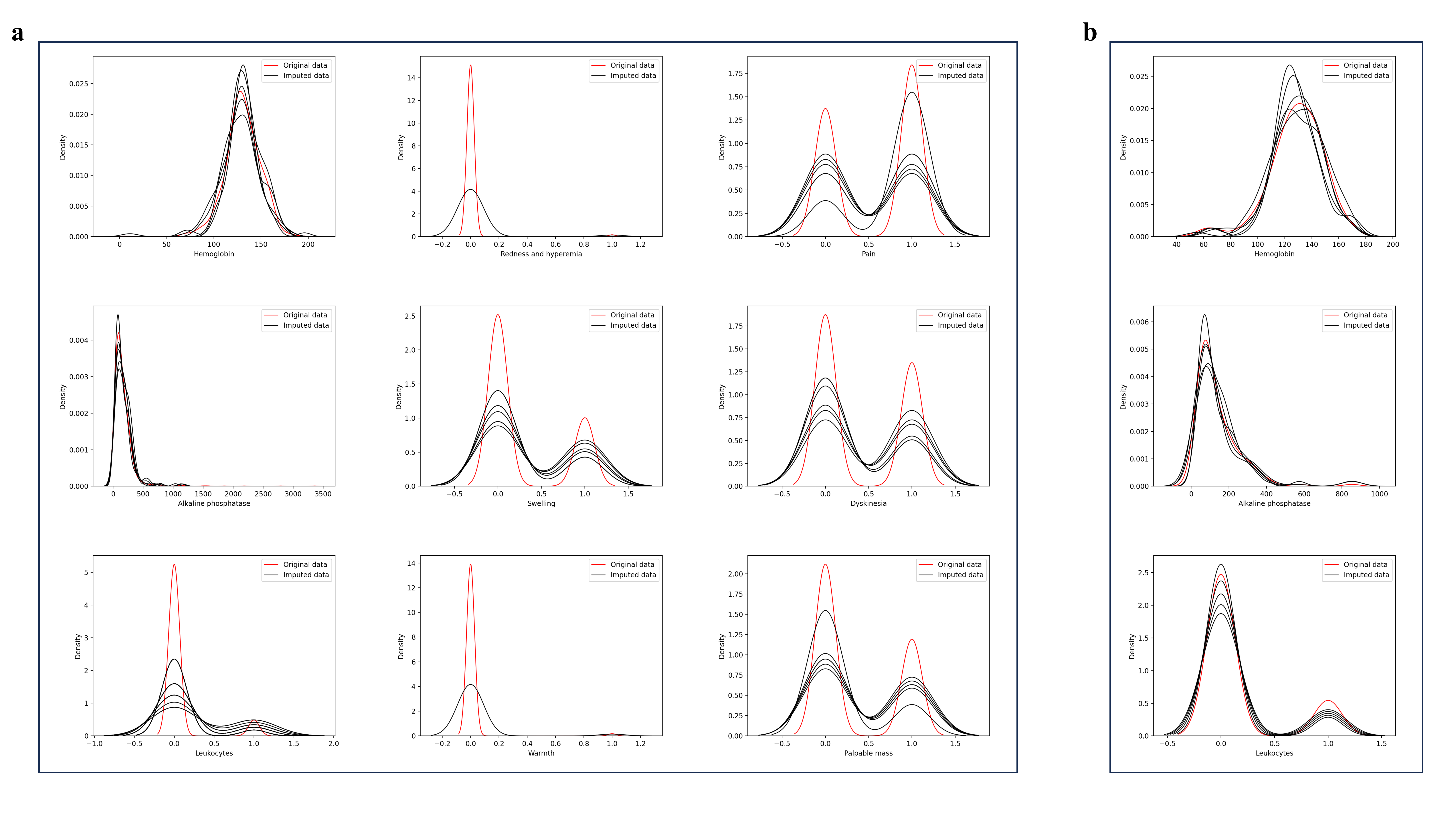
**

**Fig. S2** The variable distribution of each imputed clinical data compared to the original data on the internal (**a**) and external (**b**) datasets


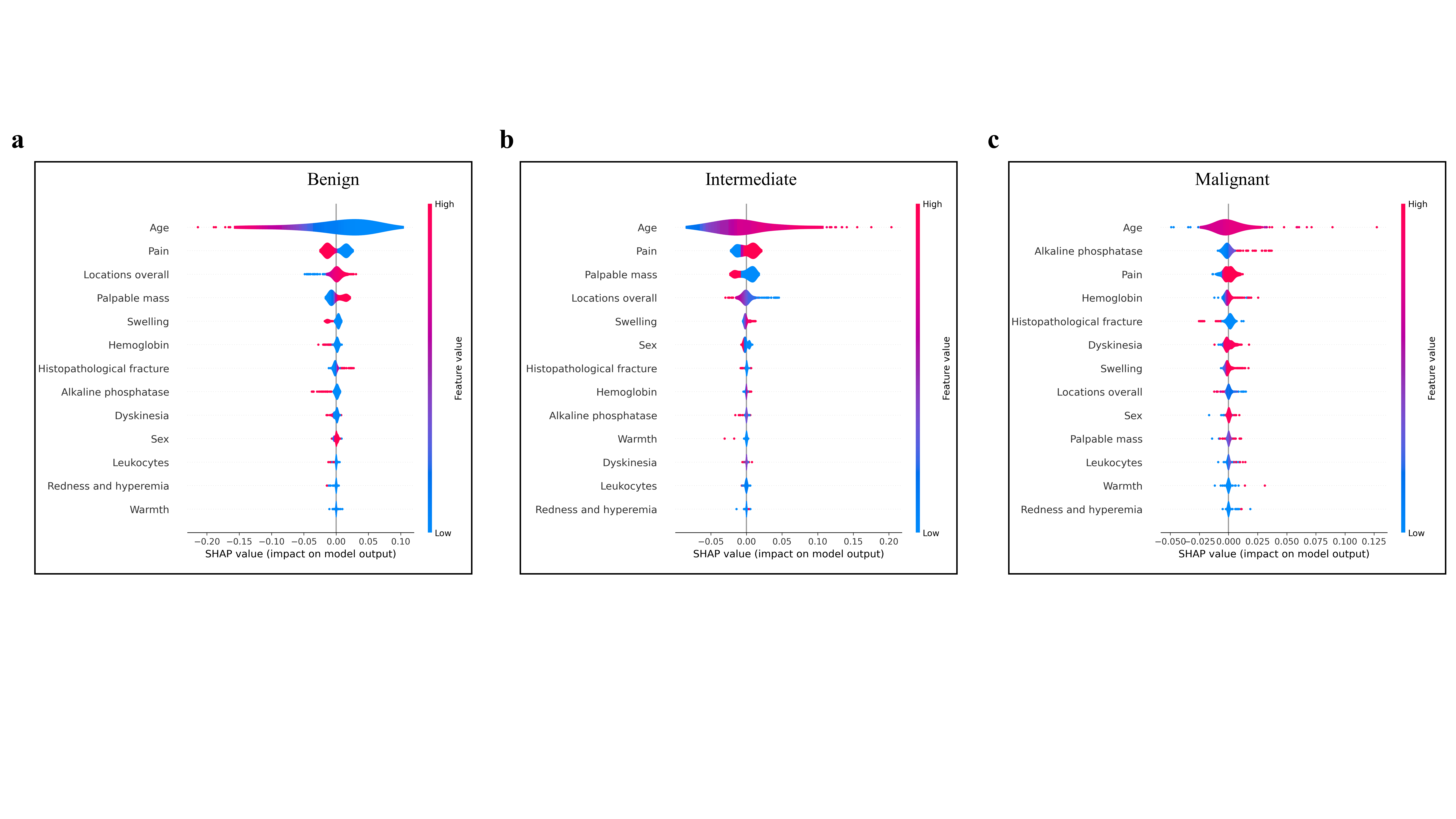


**Fig. S3** SHAP violin plots showing the impact of clinical characteristics on the model's predictions for classifying benign (**a**), intermediate (**b**), and malignant (**c**) PBTs on the external test set. Each violin plot displays the top clinical characteristics based on their SHAP values, which represent the characteristic's contribution to the model's output. The color bar indicates the feature value, with red representing high feature values and blue representing low feature values. SHAP, Shapley Additive Explanations; PBTs, Primary bone tumors
