# Supplementary material for: A deep learning model to enhance the classification of primary bone tumors based on incomplete multimodal images in X-ray, CT, and MRI
Source: Cancer Imaging. 2024 Oct 10;24:135. doi: 10.1186/s40644-024-00784-7 (PMC11468403; doi:10.1186/s40644-024-00784-7)
Supplement: Supplementary file 2 — Supplementary Material 2. [file 40644_2024_784_MOESM2_ESM.docx]

**Supplementary Material 2**

Checklist for Artificial Intelligence in Medical lmaging (CLAIM)

| **Section/Topic** | **No** | **Item** | **Yes/No** |
| --- | --- | --- | --- |
| TITLE or ABSTRACT |  |  | ☑ |
|  | 1 | Identification as a study of Al methodology, specifying the category of technology used (e.g., deep learning) | ☑ |
| ABSTRACT |  |  |  |
|  | 2 | Structured summary of study design, methods, results, and conclusions | ☑ |
| INTRODUCTION |  |  | ☑ |
|  | 3 | Scientific and clinical background, including the intended use and clinical role of the Al approach | ☑ |
|  | 4 | Study objectives and hypotheses | ☑ |
| METHODS |  |  |  |
| Study Design | 5 | Prospective or retrospective study | ☑ |
|  | 6 | Study goals, such as model creation, exploratory study, feasibility study, noninferiority trial | ☑ |
| Data | 7 | Data sources | ☑ |
|  | 8 | Eligibility criteria: how, where, and when potentially eligible participants or studies were identified (e.g. symptoms, results from previous tests, inclusion in registry, patient-care setting, location, dates) | ☑ |
|  | 9 | Data preprocessing steps | ☑ |
|  | 10 | Selection of data subsets, if applicable | ☑ |
|  | 11 | Definitions of data elements, with references to common data elements | ☑ |
|  | 12 | De-identification methods | ☑ |
|  | 13 | How missing data were handled | ☑ |
| Ground Truth | 14 | Definition of ground truth reference standard, in sufficient detail to allow replication | ☑ |
|  | 15 | Rationale for choosing the reference standard (if alternatives exist) | ☑ |
|  | 16 | Source of ground truth annotations; qualifications and preparation of annotators | ☑ |
|  | 17 | Annotation tools | ☑ |
|  | 18 | Measurement of inter- and intrarater variability; methods to mitigate variability and/or resolve discrepancies | ☑ |
| Data Partitions | 19 | Intended sample size and how it was determined | ☑ |
|  | 20 | How data were assigned to partitions; specify proportions | ☑ |
|  | 21 | Level at which partitions are disjoint (eg, image, study, patient, institution) | ☑ |
| Model | 22 | Detailed description of model, including inputs, outputs, all intermediate layers and connections | ☑ |
|  | 23 | Software libraries, frameworks, and packages | ☑ |
|  | 24 | Initialization of model parameters (eg, randomization, transfer learning) | ☑ |
| Training | 25 | Details of training approach, including data augmentation, hyperparameters, number of models trained | ☑ |
|  | 26 | Method of selecting the final model | ☑ |
|  | 27 | Ensembling techniques, if applicable | ☑ |
| Evaluation | 28 | Metrics of model performance | ☑ |
|  | 29 | Statistical measures of significance and uncertainty (e.g., confidence intervals) IEE | ☑ |
|  | 30 | Robustness or sensitivity analysis | ☑ |
|  | 31 | Methods for explainability or interpretability (e.g., saliency maps) and how they were validated | ☒ |
|  | 32 | Validation or testing on external data | ☑ |
| RESULTS |  |  |  |
| Data | 33 | Flow of participants or cases, using a diagram to indicate inclusion and exclusion | ☑ |
|  | 34 | Demographic and clinical characteristics of cases in each partition | ☑ |
| Model performance | 35 | Performance metrics for optimal model(s) on all data partitions | ☑ |
|  | 36 | Estimates of diagnostic accuracy and their precision (such as 95% confidence intervals) I | ☑ |
|  | 37 | Failure analysis of incorrectly classified cases | ☑ |
| DISCUSSION |  |  | ☑ |
|  | 38 | Study limitations, including potential bias, statistical uncertainty, and generalizability | ☑ |
|  | 39 | Implications for practice, including the intended use and/or clinical role | ☑ |
| OTHER  INFORMATION |  |  |  |
|  | 40 | Registration number and name of registry | ☒ |
|  | 41 | Where the full study protocol can be accessed | ☑ |
|  | 42 | Sources of funding and other support; role of funders | ☑ |
